# Machine Learning-Enabled 30-Day Readmission Model for Stroke Patients

**DOI:** 10.3389/fneur.2021.638267

**Published:** 2021-03-31

**Authors:** Negar Darabi, Niyousha Hosseinichimeh, Anthony Noto, Ramin Zand, Vida Abedi

**Affiliations:** ^1^Department of Industrial and Systems Engineering, Virginia Tech, Falls Church, VA, United States; ^2^Geisinger Neuroscience Institute, Geisinger Health System, Danville, PA, United States; ^3^Department of Molecular and Functional Genomics, Geisinger Health System, Danville, PA, United States; ^4^Biocomplexity Institute, Virginia Tech, Blacksburg, VA, United States

**Keywords:** ischemic stroke, 30-day readmissions, machine learning, statistical analysis, patient readmission

## Abstract

**Background and Purpose:** Hospital readmissions impose a substantial burden on the healthcare system. Reducing readmissions after stroke could lead to improved quality of care especially since stroke is associated with a high rate of readmission. The goal of this study is to enhance our understanding of the predictors of 30-day readmission after ischemic stroke and develop models to identify high-risk individuals for targeted interventions.

**Methods:** We used patient-level data from electronic health records (EHR), five machine learning algorithms (random forest, gradient boosting machine, extreme gradient boosting–XGBoost, support vector machine, and logistic regression-LR), data-driven feature selection strategy, and adaptive sampling to develop 15 models of 30-day readmission after ischemic stroke. We further identified important clinical variables.

**Results:** We included 3,184 patients with ischemic stroke (mean age: 71 ± 13.90 years, men: 51.06%). Among the 61 clinical variables included in the model, the National Institutes of Health Stroke Scale score above 24, insert indwelling urinary catheter, hypercoagulable state, and percutaneous gastrostomy had the highest importance score. The Model's AUC (area under the curve) for predicting 30-day readmission was 0.74 (95%CI: 0.64–0.78) with PPV of 0.43 when the XGBoost algorithm was used with ROSE-sampling. The balance between specificity and sensitivity improved through the sampling strategy. The best sensitivity was achieved with LR when optimized with feature selection and ROSE-sampling (AUC: 0.64, sensitivity: 0.53, specificity: 0.69).

**Conclusions:** Machine learning-based models can be designed to predict 30-day readmission after stroke using structured data from EHR. Among the algorithms analyzed, XGBoost with ROSE-sampling had the best performance in terms of AUC while LR with ROSE-sampling and feature selection had the best sensitivity. Clinical variables highly associated with 30-day readmission could be targeted for personalized interventions. Depending on healthcare systems' resources and criteria, models with optimized performance metrics can be implemented to improve outcomes.

## Introduction

Hospital readmissions impose a substantial financial burden, costing Medicare about $26 billion annually (1). Centers for Medicare and Medicaid Services (CMS) has made reducing 30-day readmission rates a national healthcare reform goal (2) as a way to improve hospital care. Reducing readmissions after stroke could lead to improved quality of care especially since stroke is associated with a high rate of readmission (3).

Studies have found that stroke severity (3, 4), being discharged to skilled nursing, intermediate care facility, hospice, or left against doctor's advice (2, 3, 5–7), being enrolled in Medicaid/Medicare (4, 6, 8, 9), and being married (5) were associated with higher readmissions. A longer length of hospital stay was associated with lower readmissions among stroke patients (5). Heart failure (2, 6, 9), coronary artery disease (10, 11), and dysphagia (4) were also correlated with stroke readmissions. Additionally, patients with anemia, dementia, malnutrition, and diabetes were more likely to be readmitted within 30-day (2, 5, 6, 9).

However, previous studies [Supplementary Table I (12)] included a limited number of variables and used logistic regression which restricts the number of included interactions among the variables (13, 14), thus limiting the model performance. Machine learning (ML), more appropriate for high-dimensional datasets (15, 16), has been successfully applied for predicting readmissions after heart failure (17–19), heart attack (20), and other causes of readmissions (21, 22). The goal of this study was to develop prediction models of 30-day readmission among patients with ischemic stroke and identify associated predictors for the development of a more targeted intervention.

## Methods

### Study Population

This study was based on the retrospective analysis of prospectively collected data from acute ischemic stroke (AIS) patients at two tertiary centers in Geisinger Health System between January 1, 2015, and October 7, 2018 (23). The data were extracted from electronic health records and de-identified. As a part of the de-identification process, the age of patients older than 89 years old was masked. Patients younger than 18 years of age were excluded from this study. Patients with transient ischemic attack were not included in this study due to the high rate of overdiagnosis (24). The study was reviewed and approved by the Geisinger Institutional Review Board to meet “Non-human subject research,” for using de-identified information.

### Data Elements

The outcome measure was hospital readmission within 30-day after discharge among patients with AIS. Independent variables included patient age, length of stay (LOS), gender, marital status (married, single, and previously married), and the National Institutes of Health Stroke Scale (NIHSS). The types of health insurance at the time of first admission (Medicare, Medicaid, private, direct employer contract, self-pay, worker compensation, and other government payers) were also included. Other variables in this study were six discharge destinations (discharged to the home health organizations; discharged to home, court, or against medical advice; discharged to hospice-home/hospice-medical facility; discharged or transferred to other facilities; discharged or transferred to Skilled Nursing Facility, SNF; discharged or transferred to another rehab facility), and five clinical interventions (intravenous thrombolysis; insert indwelling urinary catheter; endotracheal tube; percutaneous gastrostomy; and hemodialysis). In addition, a total of 47 comorbidities were included (see Table 1).

**Table 1:** Descriptive statistics of variables.

**Variables**		**Missing**	**Not Readmitted (*n* = 2883)**	**Readmitted (*n* = 301)**	***t* statistics**	***P*-value**
	Age (y), Mean (SD)	12	71.10(13.90)	71.50(12.90)	−0.52	0.60
	LOS^a^ (d), Median (IQR)	319	3 (1, 76)	4 (1, 24)	−4.03	0.00
	All gender	12	2873	299	–	–
Gender, *n* (%)	Female		1406 (48.80)	155 (51.50)	−0.90	0.39
	Male		1467 (50.90)	144 (47.80)	1.00	0.31
	Total	12	2873	299		
Marital Status, *n* (%)	Married		1349 (46.80)	124 (41.20)	1.85	0.06
	Single		425 (14.70)	45 (15.00)	−010	0.92
	Previously married		1099 (38.10)	130 (43.20)	−1.72	0.08
	0 to above 24	2545	580	59	–	–
NIHSS Score, *n* (%)	0 to 4		330 (11.40)	31 (10.30)	0.60	0.55
	5 to 11		150 (5.20)	17 (5.60)	−0.33	0.74
	12 to 23		75 (2.60)	10 (3.30)	−0.74	0.46
	Above 24		25 (0.90)	1 (0.30)	0.98	0.33
	Total	-	291	36	–	–
Procedures, *n* (%)	Intravenous thrombolysis		71 (3.00)	2 (0.80)	2.06	0.04
	Insert indwelling urinary catheters		3 (0.10)	1 (0.30)	−1.07	0.28
	Insert endotracheal tube		148 (5.10)	10 (3.30)	1.35	0.17
	Percutaneous gastrostomy		42 (1.50)	16 (5.40)	−4.81	0.00
	Hemodialysis		27 (0.90)	7 (2.30)	−2.25	0.02
	All centers	-	2883	301	–	–
Hospital, *n* (%)	GMC^b^		1784 (61.90)	176 (58.50)	1.08	0.27
	GWV^c^		1099 (38.10)	125 (41.50)	−1.16	0.25
	Total	492	2418	274	–	–
Discharge Status, *n* (%)	Discharged to home health organization		346 (12.00)	37 (12.30)	−0.15	0.88
	Discharged to home, court, or against medical advice		902 (31.30)	57 (18.90)	4.46	0.00
	Discharged to hospice-home/hospice-medical facility		82 (2.80)	4 (1.30)	1.54	0.12
	Discharged/transferred to other facilities		27 (0.90)	2 (0.70)	0.47	0.63
	Discharged/transferred to *SNF*^*d*^		447 (15.50)	91 (30.20)	−6.53	0.00
	Discharged/transferred to another rehab facility		614 (21.30)	83 (27.60)	−2.51	0.01
	Total	51	2836	297	–	–
Payer, *n* (%)	Direct employer contract		65 (2.30)	9 (3.00)	−0.80	0.42
	Medicaid		216 (7.50)	22 (7.30)	0.11	0.91
	Medicare		2037 (70.70)	230 (76.40)	−2.10	0.03
	Other government payers		58 (2.00)	4 (1.30)	0.81	0.41
	Private		425 (14.70)	30 (1.00)	2.25	0.02
	Self-pay		32 (1.10)	1 (0.30)	1.27	0.20
	Workers compensation		3 (0.10)	1 (0.30)	−1.06	0.29
Diagnoses, *n* (%)	Anemia	-	319 (13.70)	67 (26.40)	−5.42	0.00
	Atrial fibrillation		691 (29.40)	93 (36.30)	−2.29	0.02
	Anxiety disorders		328 (14.00)	53 (20.70)	−2.90	0.00
	Cerebral arterial dissection		23 (1.00)	6 (2.30)	−1.98	0.05
	Coronary artery disease		684 (29.10)	77 (30.10)	−0.32	0.75
	Delirium		44 (1.90)	12 (4.70)	−2.95	0.00
	Dementia		222 (9.50)	35 (13.70)	−2.15	0.03
	Diabetes		642 (27.50)	86 (33.90)	−2.13	0.03
	Dysphagia		150 (6.40)	29 (11.30)	−2.97	0.00
	Heart failure		426 (18.30)	64 (25.20)	−2.67	0.00
	Hypercoagulable state		17 (0.70)	7 (2.70)	−3.20	0.00
	Hypertension		1308 (56.10)	131 (51.60)	1.38	0.17
	Hypotension		55 (2.30)	13 (5.10)	−2.61	0.00
	Kidney disease		670 (28.50)	101 (39.50)	−3.65	0.00
	Malignancy		286 (12.20)	47 (18.40)	−2.82	0.00
	Malnutrition		105 (4.50)	32 (12.50)	−5.49	0.00
	Migraine		69 (2.90)	13 (5.10)	−1.86	0.06
	Overweight		58 (2.50)	16 (6.20)	−3.46	0.00
	Tobacco use		1171 (49.90)	143 (55.90)	−1.83	0.07
	Venous thrombosis		81 (3.40)	18 (7.00)	−2.85	0.00
	Acute myocardial infarction		36 (1.50)	9 (3.50)	−2.31	0.02
	Alcohol use		95 (4.00)	10(3.90)	0.11	0.91
	Arrhythmias		99 (4.20)	15 (5.90)	−1.22	0.22
	Blindness		14 (0.60)	1 (0.40)	0.41	0.68
	Cardiac valvular disease		159 (6.80)	22 (8.60)	−1.09	0.27
	Cardiomyopathy		131 (5.60)	23 (9.00)	−2.20	0.03
	Cerebral atherosclerosis		93 (4.00)	13 (5.10)	−0.86	0.39
	Chronic kidney disease		583 (25.00)	89 (35.00)	−3.47	0.00
	Chronic liver disease		49 (2.10)	2 (0.80)	1.43	0.15
	Chronic lung disease		476 (20.30)	69 (27.00)	−2.50	0.01
	Dysautonomia		16 (0.70)	2 (0.80)	−0.18	0.85
	Hyperlipidemia		1537 (65.40)	177 (69.10)	−1.19	0.23
	Intracerebral hemorrhage		521 (22.20)	51 (19.90)	0.83	0.41
	Inflammatory disorders		66 (2.80)	5 (2.00)	0.80	0.42
	Mood disorders		372 (15.80)	58 (22.70)	−2.79	0.00
	Non-compliance		107 (4.60)	12 (4.70)	−0.09	0.92
	Normal weight		44 (1.90)	11 (4.30)	−2.56	0.01
	Obese		466 (19.80)	62 (24.20)	−1.65	0.09
	Palliative care on board		254 (10.80)	12 (4.70)	3.08	0.00
	Peripheral vascular disease		125 (5.30)	17 (6.60)	−0.88	0.38
	Respiratory failure		164 (7.00)	19 (7.40)	−0.26	0.79
	Seizure disorders		96 (4.10)	15 (5.90)	−1.33	0.18
	Sleep apnea		216 (9.20)	25 (9.80)	−0.30	0.76
	Systemic infection		63 (2.70)	16 (6.20)	−3.17	0.00
	Thyroid disease		445 (18.90)	54 (21.10)	−0.83	0.41
	Underweight		34 (1.40)	9 (3.50)	−2.47	0.01
	Use of steroids		72 (3.10)	11 (4.30)	−1.06	0.29
	Year, Median (IQR)	–	2016 (2015, 2018)	2016 (2015, 2018)	−0.36	0.72

a *length of stay*;

b *geisinger medical center*,

c *Geisinger wyoming valley*,

d *skilled nursing facility*.

### Data Processing, Feature Selection, and Sampling

Pearson's correlation coefficient was applied to continuous variables to identify those with high collinearity. The correlation matrix between all the predictors along with a list of correlations above 30 and 50% is provided in Supplementary Figure I and Supplementary Table II (12), respectively. The complete list of variables along with their descriptive statistics and level of missingness was provided in Table 1. Student's *t*-test was applied to identify the significant difference between two groups of patients (i.e., readmitted and not readmitted) for each predictor and the test statistics and *P*-values were reported in Table 1.

**Figure 1:**
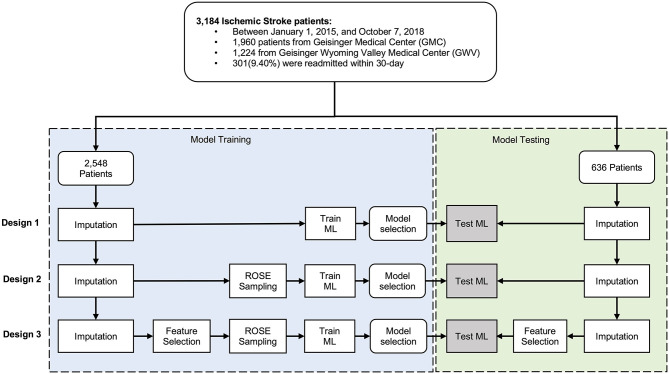
Data processing flowchart.

Some of the variables were suffering from missing observations (see Table 1). Imputation, using Multivariate Imputation by Chained Equations (MICE) package in R (25), was performed separately on the training and testing sets to ensure an unbiased evaluation of the final model. For the variables with high missingness, we performed an assessment of the distribution of the variable before and after imputation. We used two sets of variables, set one was the comprehensive set including all the variables, and set two included variables selected based on data-driven feature selection, where variables with high collinearity were removed. We used the random forest classification algorithm by Boruta package in RStudio (26) for our data-driven feature selection. Further, to avoid the poor performance of the minority class compared to the dominant class, we applied an adaptive sampling strategy, where we balanced the dataset by applying the Random Over-Sampling Examples (ROSE) algorithm on the minority class (27). The data cleaning and preparation were performed in STATA 14.0 (28) and the analyses were performed using R 3.6.0 (29) in R studio. Figure 1 shows the processing and modeling pipeline.

### Model Development

The de-identified dataset was randomly split into the train set (80%) and test set (20%). We developed models to predict 30-day readmission of ischemic stroke using the training dataset and used ten-fold cross-validation to select the best performing model. Overall, we built fifteen models – based on five different algorithms – following three study designs (Design 1, 2, and 3, see Figure 1). The five algorithms included logistic regression (LR), random forest (RF), gradient boosting machine (GBM), extreme gradient boosting (XGBoost), and support vector machines (SVM). Parameter tuning was performed by an automatic grid search with ten different values to randomly try for each algorithm parameter. All the hyperparameter evaluation and model development were performed using the Caret package in R Studio (30). We ran the SVM with and without normalization of the dataset. In normalization, we scaled the data to calculate the standard deviation for an attribute and divided each value by that standard deviation. Then we centered the data to calculate the mean for an attribute and subtracted it from each value. The performance measures of the models were evaluated using the 20% test set. To compare the performance of the applied models, we calculated the area under the receiver operating characteristic curve (AUC). We also used other performance measures such as sensitivity or recall, specificity, and positive predictive value (PPV) as well as training time.

## Results

### Study Design and Population Characteristics

A total number of 3,184 AIS patients [1,960 patients from Geisinger Medical Center (GMC) and 1,224 from Geisinger Wyoming Valley Medical Center (GWV)] were included in this study.

Among 3,184 patients with ischemic stroke, 301(9.40%) were readmitted within 30-day. The train set and test set included 2,548 (80%) and 636 (20%) patient-level observations, respectively. In Table 1, the patients were compared based on diverse characteristics including demographic characteristics, medical history prior to the ischemic stroke event, and stroke severity using the NIHSS score. Continuous variables were presented as mean and standard deviation and as median with interquartile range (IQR). The average age of patients was 71 (interquartile range, IQR: 18–89) and 1,611(50.60%) patients were men. There was a significant difference between patients who were readmitted and those who were not in terms of median LOS, being married or previously married, discharged to SNF or against medical advice, and having Medicare or private insurance.

### Models Can Be Trained to Predict 30-Day Readmission Using EHR

The performance metrics—AUC and its 95% confidence interval (CI), sensitivity, specificity, PPV, and the training time —for all the 15 models with and without ROSE-sampling (Design 2, and 1), and with feature selection and ROSE-sampling (Design 3) were reported in Table 2. The CIs for the test sets were calculated using bootstrapping. We also provided the confusion matrices of all 15 models in Supplementary Table V. The results showed that applying ROSE for addressing class imbalance during the model training improved the AUC, PPV, and specificity of models during the testing phase. However, feature selection did not improve the results [see Table 2, and Supplementary Figures II, III (12)]. Feature selection was performed using the Boruta package which reduced the number of features from 52 to 14 [see green variables in Supplementary Figure IV (12)]. These 14 attributes were used in the third design while all features were included in the other designs.

**Table 2:** Performance metrics for machine learning models.

	**Train set**		**Test set**				
**Method**	**AUC**	**Training Time (s)**	**AUC**	**95% CI for AUC**	**Sensitivity**	**Specificity**	**PPV**
	**No feature selection and sampling (Design 1)**						
LR	0.76	3	0.60	(0.52, 0.67)	0.32	0.86	0.19
RF	0.82	42	0.57	(0.50, 0.64)	0.09	0.93	0.12
GBM	0.70	48	0.68	(0.52, 0.76)	0.23	0.95	0.33
XGBoost	0.76	1,752	0.62	(0.56, 0.69)	0.30	0.88	0.21
SVM	0.98	990	0.62	(0.56, 0.70)	0.30	0.86	0.18
	**With ROSE-sampling (Design 2)**						
LR	0.74	3	0.63	(0.55, 0.70)	0.38	0.72	0.12
RF	0.74	33	0.67	(0.51, 0.76)	0.09	0.97	0.26
GBM	0.74	48	0.70	(0.61, 0.75)	0.09	0.98	0.45
XGBoost	0.76	2,340	0.74	(0.64, 0.78)	0.20	0.98	0.43
SVM	0.83	1,689	0.67	(0.59, 0.74)	0.38	0.89	0.27
	**With feature selection and ROSE-sampling (Design 3)**						
LR	0.70	3	0.64	(0.56, 0.72)	0.53	0.69	0.15
RF	0.70	12	0.65	(0.56, 0.70)	0.30	0.89	0.24
GBM	0.69	30	0.66	(0.58, 0.74)	0.17	0.95	0.26
XGBoost	0.70	2,130	0.65	(0.56, 0.73)	0.17	0.95	0.27
SVM	0.72	960	0.64	(0.56, 0.72)	0.42	0.77	0.16

**Figure 2:**
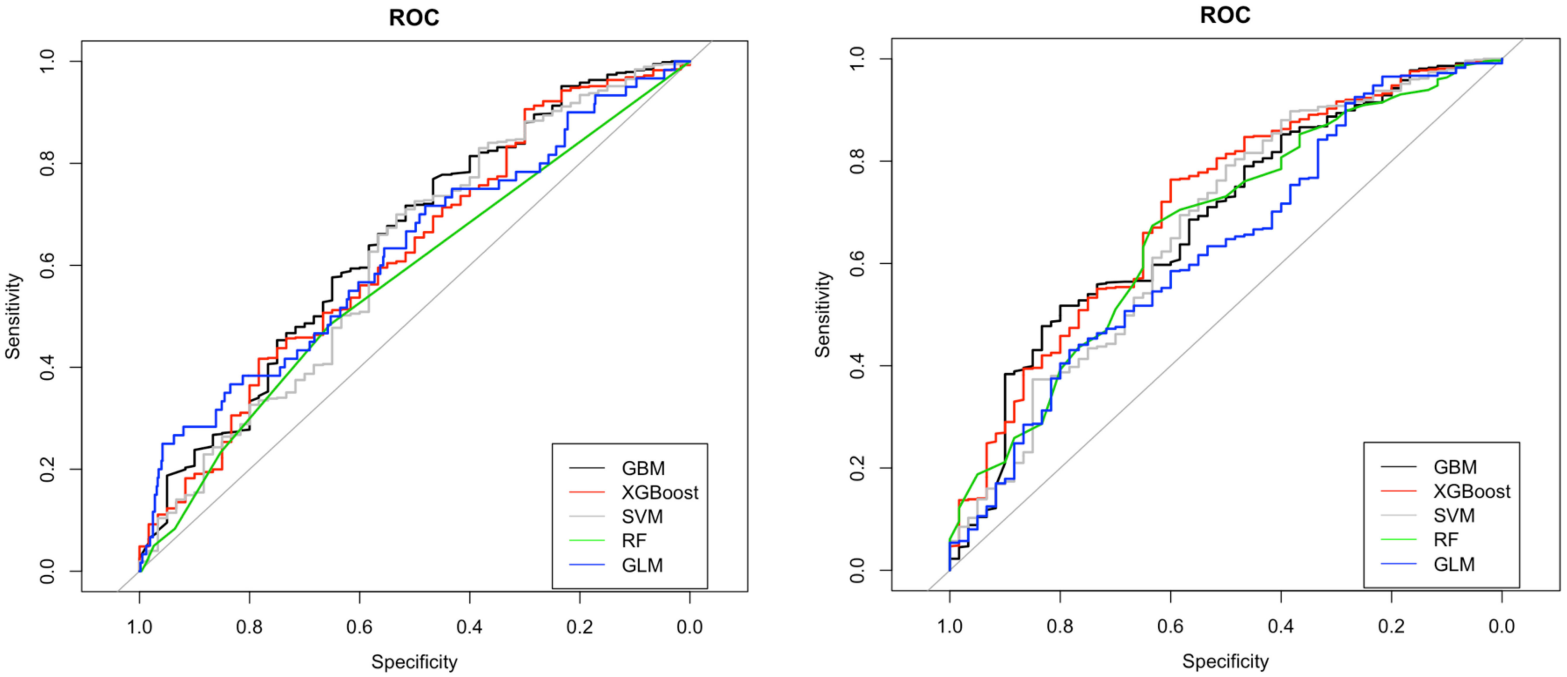
ROC curves for machine learning models with (bottom) and without (top) ROSE-sampling. GBM, gradient boosting machine; XGBoost, extreme gradient boosting; SVM, support vector machines; RF, random forest; and GLM, generalized linear model with logit link which is logistic regression in our study.

The ROC curves for LR, RF, GBM, XGBoost, and SVM without feature selection and sampling (Design 1) and with ROSE-sampling (Design 2) were shown in the top and bottom side of Figure 2 accordingly. In the absence of sampling and feature selection, GBM provided the highest AUC (0.68), specificity (0.95), and PPV (0.33) when compared to the other models (Figure 2 and Table 2). However, the best AUC (0.74), PPV (0.43), and specificity (0.98) were reached when ROSE-sampling was applied. The optimal model parameters for ROSE-sampled XGBoost were max-depth = 4, subsample = 0.50, colsample_bytree = 0.80, gamma = 0, and min_child_weight = 10. In terms of AUC, specificity, and PPV, the LR in Design 2 had poor performance compared to XGBoost and GBM models. However, LR with feature selection and ROSE-sampling (Design 3) provided the highest sensitivity (0.53) relative to other models. We also performed SVM with normalized data and the results are provided in Supplementary Table IV.

The training times for LR, RF, and GBM were faster when compared to models based on XGBoost and SVM (see Table 2). The model training was performed using MacBook Pro14,2, four thunderbolt 3 ports with 3.1 GHz Dual-Core Intel Core i5, and 8 GB memory. Overall, the addition of the sampling step increased the training time, while having fewer features resulted in faster training as expected.

### NIHSS, Insert Indwelling Urinary Catheter, Hypercoagulable State, and Percutaneous Gastrostomy Are the Top Predictors of 30-Day Readmission

Using XGBoost in Design 2, the best predictive model, we identified the most important predictors of 30-day readmission. According to the variable importance scores for XGBoost in Design 2 (Table 3), the top 10 predictors of 30-day readmission were NIHSS above 24, insert indwelling urinary catheter, hypercoagulable state, percutaneous gastrostomy, using workers compensation as insurance, hemodialysis, overweight, cerebral arterial dissection, malnutrition, intravenous thrombolysis, and venous thrombosis.

**Table 3:** Variable importance scores of the XGBoost model with ROSE-sampling (Design 2).

**No.**	**Variable**	**Importance Score (out of 100)**
1	NIHSS above 24	100.00
2	Insert Indwelling Urinary Catheter	89.94
3	Hypercoagulable State	61.95
4	Percutaneous Gastrostomy	40.04
5	Payer Workers Compensation	37.78
6	Hemodialysis	35.13
7	Overweight	33.35
8	Cerebral Arterial Dissection	30.61
9	Malnutrition	25.74
10	intravenous thrombolysis	24.15
11	Venous Thrombosis	22.94
12	Discharged to Hospice-Home or Hospice-Medical Facility	17.15
13	Palliative Care on Board	14.92
14	Delirium	14.54
15	Payer Self-Pay	14.42

We also reported the result of LR in the third design. In the latter, the multicollinearity was addressed by feature selection (Table 4). The odds ratios (OR), log odds, 95% CI, and *P*-values were reported in this table. This analysis revealed that being discharged to SNF, malignancy, and malnutrition were significantly associated with stroke readmission within 30-day (*p*-value < 0.0001). Also, being discharged to a rehabilitation facility and stroke severity above twelve were significantly associated with 30-day readmission at 0.001 significance level.

**Table 4:** Logistic regression results for predictors of 30-day readmission in ischemic stroke patients (Design 3).

**Variables**	**OR**	**Log Odds**	**95% CI (2.5%, 97.5%)**		***P*-value**
(Intercept)	0.25	−1.39	−2.55	−0.30	0.01
Age	0.99	−0.01	−0.03	0.00	0.07
Discharged to home health organization	1.61	0.47	−0.01	0.94	0.05
Discharged to hospice-home or hospice-medical facility	0.44	−0.83	−2.04	0.28	0.16
Discharged/transferred to other facilities	0.49	−0.70	−2.62	0.66	0.38
Discharged/transferred to another rehab facility	1.79	0.58	0.17	0.99	0.01
Discharged/transferred to SNF	2.77	1.02	0.56	1.48	0.00
Medicaid	0.41	−0.89	−1.79	0.07	0.06
Medicare	0.57	−0.55	−1.31	0.30	0.17
Other government payers	0.39	−0.93	−2.33	0.32	0.16
Private insurance	0.40	−0.91	−1.75	−0.00	0.04
Self-pay	0.29	−1.23	−4.21	0.59	0.27
Workers compensation	1.82	0.60	−2.59	3.04	0.65
Chronic kidney disease	1.28	0.24	−0.36	0.89	0.44
Hypercoagulable state	3.09	1.13	0.18	1.99	0.01
Kidney disease	1.15	0.14	−0.49	0.72	0.65
Malignancy	2.10	0.74	0.37	1.09	0.00
Malnutrition	2.51	0.92	0.39	1.42	0.00
Palliative care on board	1.13	0.13	−0.73	0.90	0.76
Respiratory failure	0.74	−0.30	−1.06	0.39	0.41
Underweight	1.00	0.00	−1.01	0.91	0.99
Insert endotracheal tube	0.86	−0.15	−1.11	0.74	0.76
Percutaneous gastrostomy	1.39	0.33	−0.51	1.09	0.42
NIHSS 12 to 23	1.76	0.57	0.14	0.99	0.01
NIHSS 5 to 11	0.65	−0.43	−0.79	−0.08	0.02
NIHSS above 24	0.17	−1.77	−3.25	−0.65	0.01

## Discussion

We have taken a comprehensive approach to identify and prioritize factors associated with 30-day readmissions after ischemic stroke. We aimed to find the most effective predictive model by comparing the results of different ML techniques and LR. There have been multiple readmission studies that developed predictive models for the chances of 30-day readmission in stroke patients. However, most of these models used LR (31) which limits the inclusion of higher-order interactions among variables and does not perform well in the presence of collinearity. Also, many studies considered readmission after 90 days or 1 year as a dependent variable which is a long follow-up period, as CMS penalizes healthcare systems for readmission under 30 days. In this study, we addressed these gaps and improved the prediction performance of readmissions in stroke patients using a wide range of potential risk factors and the proper ML techniques. Our results show that depending on the resources and criteria of healthcare systems, a predictive model with optimized performance metrics can be used to improve decision making.

### Machine Learning-Based Models Can Be Trained to Predict 30-Day Readmission

The results of this study indicate that ML-based models can be designed to predict 30-day readmission after stroke using structured data from EHR. ML algorithms can include higher-order interactions among variables, handle multicollinearity, and improve readmission predictions when applied to large and high-dimensional datasets (15). This study was the first in predicting the associated variables of 30-day ischemic stroke readmission using ML techniques. Our findings indicated that the best performance in terms of AUC, specificity, and PPV was obtained when XGBoost was used with ROSE-sampling.

Past studies that used ML techniques to improve the prediction power, either performed their analysis on readmission more than 30-day or studied other causes of readmission such as heart failure (17, 18, 21). However, our best performing model (XGBoost in Design 2) provides higher AUC and PPV compared to these studies [See Supplementary Table III (12)].

### Clinical Features Highly Associated With 30-Day Readmission

The results of our best performing model (XGBoost in Design 2) showed that NIHSS score above 24, insert indwelling urinary catheter, hypercoagulable state, percutaneous gastrostomy, and insurance type are among factors with the highest importance. The common significant predictors of the 30-day readmission in both XGBoost in Design 2 and LR in Design 3 included NIHSS score above 24, hypercoagulable state, and malnutrition. Since NIHSS is an important variable and this variable also suffered from high missingness, we assessed its distribution before and after imputation for both train and test sets. Our results corroborate that the distribution of this variable remains the same after applying imputation (see Supplementary Table VI, Supplementary Figures V, VI).

Additionally, malignancy, NIHSS scores between 5 and 23, private insurance type, and being discharged to a rehabilitation facility or SNF were only significant in the LR, and they had low importance scores in the XGBoost model. Among all variables, stroke severity and malnutrition were found significant predictors of 30-day readmission in ischemic stroke patients in past studies and our results corroborated the previous findings (2–6, 9).

It has been shown in previous studies that heart failure and being Medicare or Medicaid user were significantly correlated with 30-day readmission (2, 4, 6, 8, 9). However, we found no evidence in favor of these assertions. Past studies provided mixed results on the importance of age, hypertension, and gender; some studies found that patients of older age were more likely to be readmitted (2, 5) while others showed that age was not a significant predictor (3, 32). Also, hypertension was found as a significant risk factor of readmission in a study (8) while in other works authors claimed that hypertension was not significantly associated with 30-day readmission (13, 32). Several studies conducted on data from Taipei, China, and Western Australia found that gender of patients was not significantly associated with the chances of being readmitted (3, 5, 32); however, studies based on U.S. data have found women were significantly at higher risk of readmission (2, 8, 13). The results of the ROSE-sampled XGBoost model indicated that age, hypertension, and gender–in this specific cohort–were not significantly associated with 30-day readmission after ischemic stroke. We have also performed a detailed analysis of our Geisinger cohort and identified that sex was not an independent risk factor for all-cause mortality and ischemic stroke recurrence (33). Finally, the identification of malnutrition provides potential new venues to improve secondary prevention and outcome (34).

### Model Performance Metrics Optimized Based on the Target Goals

According to our results, the best performing predictive model, which was ROSE-sampled XGBoost, had a 17.5% improvement in AUC compared to LR. This XGBoost performed better in comparison with other models of 30-day readmission in the literature (17, 18, 21). We improved the AUC up to 0.74 (95% CI: 0.64, 0.78) for the test set with 0.43 PPV (see Design 2 in Table 2). In the absence of sampling and feature selection, GBM returned very close AUC for the training and testing sets, corroborating that the models did not suffer from overfitting (Design 1 in Table 2). XGBoost and GBM with ROSE-sampling achieved comparable AUC for the testing and training sets, confirming that these models did not suffer from overfitting (Design 2 in Table 2). However, the SVM-based models had the largest difference between testing and training AUC, leading to the possibility of overfitting given this dataset. Overall, ML-based models such as GBM and XGBoost improved the prediction of 30-day readmission in stroke patients compared to traditional LR [see Table 2, Supplementary Figures II, III (12)]. However, LR with feature selection and ROSE-sampling provided the best sensitivity which implies that healthcare systems can choose their decision models based on their resources and criteria.

## Limitations

One of the important strengths of this study was that we analyzed a diverse list of potential predictors including an extensive number of clinical interventions and patient's comorbidities. To the best of our knowledge, this was the first attempt to apply ML techniques to predict the 30-day readmission for ischemic stroke patients. Considering a large number of included variables in our dataset, these ML techniques could include higher-order interactions among variables, and improve the prediction power when compared to LR.

Our analysis had several limitations. Although our dataset was rich in the number of variables, the number of patients was relatively small compared to the included independent variables. Therefore, the small number of observations might result in overfitting in the models. However, comparable AUC measures provided by XGBoost for the testing and training sets rule out the possibility of overfitting in this model. Another limitation of this work was missing data specifically for the NIHSS score. The most missing data points belonged to the NIHSS score before 2016 and we applied imputation to not lose any observation or cause sampling bias. Additionally, due to the unique demographic characteristics of this dataset (the majority of patients were white and from non-urban areas), the results may not be generalizable to other health systems.

## Future Directions

In this study, we only considered ischemic stroke as the cause of readmission. Therefore, future avenues of research can be done by considering other stroke types and subtypes. However, considering the size of our dataset which came from two health centers from central Pennsylvania, further work needs to focus on a larger population with diverse demographics to introduce a generalizable model. Additionally, to improve the prediction power, future studies may include the application of deep learning techniques (35) as well as the integration of features from unstructured sources such as clinical notes and imaging reports. Finally, improvement in parameter optimization, by using sensitivity analysis (SA)-based approaches (36, 37) and improving the imputation for laboratory values for EHR-mining (38) can lead to an improvement in outcome prediction models using administrative datasets. These strategies will help in model generalizability, improve patient representation, and reduce algorithmic bias.

## Conclusion

Our results showed that machine learning-based predictive models perform better than traditional logistic regression, enabling the inclusion of a more comprehensive set of variables into the model. The insights from this work can assist with the identification of ischemic stroke patients who are at higher risk of readmission for more targeted preventive strategies. Our study also indicated the importance of including multiple performance metrics for empowering the healthcare system to choose a predictive model for implementation as an assistive decision support tool into their EHR based on their resources and criteria.

## Data Availability Statement

All relevant data are available in the article/Supplementary Material. Due to privacy and other restrictions, the primary data cannot be made openly available. Deidentified data may be available subject to data-sharing agreement with Geisinger Health System. Details about requesting access to the data are available from the Geisinger's corresponding author Vida Abedi.

## Author Contributions

NH, VA, and RZ: conception and design of the study. VA and NH: supervision of the project. AN and VA acquisition of the data. ND, VA, and NH: analysis of the data. ND: implementation of the code and Drafting a significant portion of the manuscript or figures. NH, VA, ND and RZ: interpretation of the findings. VA, RZ, and NH: editing the manuscript. ND, NH, VA, RZ, and AN: participation in discussions on the model and results. All authors contributed to the article and approved the submitted version.

## Conflict of Interest

The authors declare that the research was conducted in the absence of any commercial or financial relationships that could be construed as a potential conflict of interest.

## References

[1] LaPointe J. 3 Strategies to Reduce Hospital Readmission Rates, Costs. (2018). Available online at: https://revcycleintelligence.com/news/3-strategies-to-reduce-hospital-readmission-rates-costs (accessed January 08, 2018).

[2] Lichtman JH Leifheit-Limson EC Jones SB Wang Y Goldstein LB. Preventable readmissions within 30 days of ischemic stroke among medicare beneficiaries. Stroke. (2013) 44:3429–35. 10.1161/STROKEAHA.113.003165 24172581 PMC3905749

[3] Chuang K-Y Wu S-C Ma A-HS Chen Y-H Wu C-L. Identifying factors associated with hospital readmissions among stroke patients in Taipei. J Nurs Res. (2005) 13:117–28. 10.1097/01.JNR.0000387533.07395.42 15986313

[4] Jia H Zheng Y Reker DM Cowper DC Wu SS Vogel WB. Multiple system utilization and mortality for veterans with stroke. Stroke. (2007) 38:355–60. 10.1161/01.STR.0000254457.38901.fb 17194888

[5] Wen T Liu B Wan X Zhang X Zhang J Zhou X. Risk factors associated with 31-day unplanned readmission in 50,912 discharged patients after stroke in China. BMC Neurol. (2018) 18:218. 10.1186/s12883-018-1209-y 30587162 PMC6306006

[6] Smith MA Liou J-I Frytak JR Finch MD. 30-day survival and rehospitalization for stroke patients according to physician specialty. Cerebrovasc Dis. (2006) 22:21–6. 10.1159/000092333 16567933 PMC1635546

[7] Burke JF Skolarus LE Adelman EE Reeves MJ Brown DL. Influence of hospital-level practices on readmission after ischemic stroke. Neurology. (2014) 82:2196–204. 10.1212/WNL.0000000000000514 24838793 PMC4113457

[8] Kennedy BS. Does race predict stroke readmission? An analysis using the truncated negative binomial model. J Natl Med Assoc. (2005) 97:699. 15926648 PMC2569335

[9] Smith MA Frytak JR Liou J-I Finch MD. Rehospitalization and survival for stroke patients in managed care and traditional medicare plans. Med Care. (2005) 43:902. 10.1097/01.mlr.0000173597.97232.a0 16116355 PMC1635488

[10] Heller RF Fisher JD O'Este CA Lim LLY Dobson AJ Porter R. Death and readmission in the year after hospital admission with cardiovascular disease: the hunter area heart and stroke register. Med J Aust. (2000) 172:261–5. 10.5694/j.1326-5377.2000.tb123940.x 10860090

[11] Lin H-J Chang W-L Tseng M-C. Readmission after stroke in a hospital-based registry: risk, etiologies, and risk factors. Neurology. (2011) 76:438–43. 10.1212/WNL.0b013e31820a0cd8 21209374

[12] Supplemental Material. Available online at: https://www.ahajournals.org/journal/str (accessed March 19, 2021).

[13] Lichtman JH Leifheit-Limson EC Jones SB Watanabe E Bernheim SM Phipps MS. Predictors of hospital readmission after stroke: a systematic review. Stroke. (2010) 41:2525–33. 10.1161/STROKEAHA.110.599159 20930150 PMC3021413

[14] Ouwerkerk W Voors AA Zwinderman AH. Factors influencing the predictive power of models for predicting mortality and/or heart failure hospitalization in patients with heart failure. JACC Heart Fail. (2014) 2:429–36. 10.1016/j.jchf.2014.04.006 25194294

[15] Friedman J Hastie T Tibshirani R. The Elements of Statistical Learning. New York, NY; Springer series in statistics (2001).

[16] Noorbakhsh-Sabet N Zand R Zhang Y Abedi V. Artificial intelligence transforms the future of health care. Am J Med. (2019) 132:795–801. 10.1016/j.amjmed.2019.01.017 30710543 PMC6669105

[17] Mortazavi BJ Downing NS Bucholz EM Dharmarajan K Manhapra A Li S-X. Analysis of machine learning techniques for heart failure readmissions. Circ Cardiovasc Qual Outcomes. (2016) 9:629–40. 10.1161/CIRCOUTCOMES.116.003039 28263938 PMC5459389

[18] Golas SB Shibahara T Agboola S Otaki H Sato J Nakae T. A machine learning model to predict the risk of 30-day readmissions in patients with heart failure: a retrospective analysis of electronic medical records data. BMC Med Inform Decis Mak. (2018) 18:44. 10.1186/s12911-018-0620-z 29929496 PMC6013959

[19] Frizzell JD Liang L Schulte PJ Yancy CW Heidenreich PA Hernandez AF. Prediction of 30-day all-cause readmissions in patients hospitalized for heart failure: comparison of machine learning and other statistical approaches. JAMA Cardiol. (2017) 2:204–9. 10.1001/jamacardio.2016.3956 27784047

[20] Francisco A Stabler ME Hisey W Mackenzie TA Dorn C Denton J. Using machine learning to predict 30-day readmission of patients hospitalized with an acute myocardial infarction. Circulation. (2018) 138(Suppl. 1):A15808.

[21] Wolff P Graña M Ríos SA Yarza MB. Machine learning readmission risk modeling: a pediatric case study. Bio Med Res Int. (2019) 2019:1–19. 10.1155/2019/8532892 31139655 PMC6500604

[22] Kalagara S Eltorai AE Durand WM DePasse JM Daniels AH. Machine learning modeling for predicting hospital readmission following lumbar laminectomy. J Neurosurg Spine. (2018) 30:344–52. 10.3171/2018.8.SPINE1869 30544346

[23] Chaudhary D Khan A Shahjouei S Gupta M Lambert C Avula V. Trends in ischemic stroke outcomes in a rural population in the United States. J Neurol Sci. (2021) 422:117339. 10.1016/j.jns.2021.117339 33592506

[24] Sadighi A Stanciu A Banciu M Abedi V El Andary N Holland N. Rate and associated factors of transient ischemic attack misdiagnosis. Eneurologicalsci. (2019) 15:100193. 10.1016/j.ensci.2019.100193 31193470 PMC6529772

[25] Zhang Z. Multiple imputation with multivariate imputation by chained equation (MICE) package. Ann Transl Med. (2016) 4:30. 10.3978/j.issn.2305-5839.2015.12.63 26889483 PMC4731595

[26] Kursa MB Rudnicki WR. Feature selection with the boruta package. J Stat Softw. (2010) 36:1–13. 10.18637/jss.v036.i11

[27] Lunardon N Menardi G Torelli N. ROSE: a package for binary imbalanced learning. R journal. (2014) 6:79–89. 10.32614/RJ-2014-008

[28] STATA. STATA 14. (2015). Available online at: https://www.stata.com/stata14/ (accessed March 19, 2021).

[29] R. R 3.6.0. (2019). Available online at: https://cran.r-project.org/bin/windows/base/old/3.6.0/ (accessed April 26, 2019).

[30] Kuhn M. Building predictive models in R using the caret package. J Stat Softw. (2008) 28:1–26. 10.18637/jss.v028.i0527774042

[31] Bambhroliya AB Donnelly JP Thomas EJ Tyson JE Miller CC McCullough LD. Estimates and temporal trend for US nationwide 30-day hospital readmission among patients with ischemic and hemorrhagic stroke. JAMA Netw open. (2018) 1:e181190. 10.1001/jamanetworkopen.2018.1190 30646112 PMC6324273

[32] Lee AH Yau KK Wang K. Recurrent ischaemic stroke hospitalisations: a retrospective cohort study using Western Australia linked patient records. Eur J Epidemiol. (2004) 19:999–1003. 10.1007/s10654-004-0157-6 15648592

[33] Lambert C Chaudhary D Olulana O Shahjouei S Avula V Li J Abedi V Zand R. Sex Disparity in Long-term Stroke Recurrence and Mortality in a Rural Population in the United States. Ther Adv Neurol Disord. (2020) 13:1–12. 10.1177/1756286420971895 33414844 PMC7750897

[34] Sharma V Sharma V Khan A Wassmer DJ Schoenholtz MD Hontecillas R. Malnutrition, health and the role of machine learning in clinical setting. Front Nutr. (2020) 7:44. 10.3389/fnut.2020.00044 32351968 PMC7174626

[35] Ding L Liu C Li Z Wang Y. Incorporating artificial intelligence into stroke care and research. Stroke. (2020) 51:e351–4. 10.1161/STROKEAHA.120.031295 33106108

[36] Alam M Deng X Philipson C Bassaganya-Riera J Bisset K Carbo A. Sensitivity analysis of an enteric immunity simulator (ENISI)-based model of immune responses to helicobacter pylori infection. PLoS ONE. (2015) 10:e0136139. 10.1371/journal.pone.0136139 26327290 PMC4556515

[37] Chen X Wang W Xie G Hontecillas R Verma M Leber A. Multi-resolution sensitivity analysis of model of immune response to helicobacter pylori infection via spatio-temporal metamodeling. Front Appl Math Stat. (2019) 5:4. 10.3389/fams.2019.00004

[38] Abedi V Li J Shivakumar MK Avula V Chaudhary DP Shellenberger MJ. Increasing the density of laboratory measures for machine learning applications. J Clin Med. (2021) 10:103. 10.3390/jcm10010103 33396741 PMC7795258

