# Supplementary material for: Machine Learning-Enabled 30-Day Readmission Model for Stroke Patients
Source: Front Neurol. 2021 Mar 31;12:638267. doi: 10.3389/fneur.2021.638267 (PMC8044392; doi:10.3389/fneur.2021.638267)
Supplement: Supplementary file 1 [file Data_Sheet_1.pdf]

Supplemental Materials

Table I. Characteristics of stroke readmission studies.

Author(s)	Year	Stroke type	Number of patients	Number of variables	Study location	Method of analysis	Follow-up period
Lichtman, Leifheit-Limson (2)	2013	Ischemic	44,379	17	U.S.	Logistic regression	30-day
Chuang, Wu (3)	2005	Ischemic or hemorrhagic	489	18	Taiwan	Logistic regression	30-day
Jia, Zheng (4)	2007	Ischemic or hemorrhagic	1818	19	U.S.	Logistic regression	1 year
Wen, Liu (5)	2018	Ischemic or hemorrhagic	50,912	18	China	Logistic regression	31-day
Smith, Liou (6)	2006	Ischemic	NA	26	U.S.	Cox regression	30-day
Kennedy (8)	2005	Ischemic or hemorrhagic	38,468	21	U.S.	Truncated negative binomial regression	1 year
Smith, Frytak (9)	2005	Ischemic	9003	13	U.S.	Cox regression	30-day
Heller, Fisher (10)	2000	Ischemic or hemorrhagic	1075	7	Australia	Logistic regression	1 year
Lee, Yau (31)	2004	Ischemic	678	11	Australia	Negative binomial regression	NA

Table II. Correlations above 30% and 50% between independent variables.

Variables	Correlations 50% or more	Correlations 30% or more
Age & Medicare	0.64	0.64
CKD & Hypertension	-0.55	-0.55
CKD & Kidney Disease	0.88	0.88
Hypertension & Kidney Disease	-0.51	-0.51
Respiratory Failure & Insert Endotracheal Tube	0.54	0.54
Female & Male	-0.99	-0.99
Married & Previously Married	-0.74	-0.74
Medicare & Private	-0.64	-0.64
GMC & MWV	-1.00	-1.00
Age & Atrial Fibrillation		0.31
Age & Previously Married		0.32
Age & Discharged to Home, Court, or Against Medical Advice		-0.30
Age & Medicaid		-0.40
Age & Private		-0.45
Anxiety Disorders & Mood Disorder		0.32
Malnutrition & Normal Weight		0.47
Malnutrition & Underweight		0.41
Palliative Care on Board & Respiratory Failure		0.32
Palliative Care on Board & Insert Endotracheal Tube		0.33
Palliative Care on Board & Discharged to Hospice-Home/Hospice-Medical Facility		0.43
Year & NIHSS 0 to 4		0.36
Female & Previously Married		0.30
Married & Single		-0.39
Medicaid & Medicare		-0.45
Discharged to Home Court or Against Medical Advice & Discharged/Transferred to SNF		-0.30
Discharged to Home Court or Against Medical Advice & Discharged/Transferred to Another Rehab Facility		-0.35

Table III. Comparison between our best performing model with other 30-day readmission studies in the literature.

Article	Method	Readmission cause	Sample size (n)	PPV	Test AUC	Sensitivity	Specificity
**Best model in our study**	**XGBoost in Design 2**	**30-day ischemic stroke**	**3,184**	**0.43**	**0.74**	**0.20**	**0.98**
Mortazavi et al., 2016 (17)	RF	30-day all cause	1,004	0.22	0.63	0.61	0.61
Mortazavi et al., 2016 (17)	GBM	30-day heart failure	1,653	0.15	0.68	0.45	0.79
Golas et al. 2018 (18)	DUNs	30-day heart failure	6,369	NR	0.70	0.65	NR
Wolff et al., 2019 (21)	NB	30-day all cause	56,558	0.06	0.65	0.70	NR

NR= Not reported, RF= Random forest, GBM= Gradient Boosting Machine, DUNs= deep unified networks, NB= Naïve Bayes

Table IV. Performance metrics for machine learning models considering normalization for SVM.

	Train set		Test set				
	No feature selection and sampling (Design 1)						
Method	AUC	Training Time (sec.)	AUC	95% CI for AUC	Sensitivity	Specificity	PPV
LR	0.76	3	0.60	(0.52, 0.67)	0.32	0.86	0.19
RF	0.82	42	0.57	(0.50, 0.64)	0.09	0.93	0.12
GBM	0.70	48	0.68	(0.52, 0.76)	0.23	0.95	0.33
XGBoost	0.76	1,752	0.62	(0.56, 0.69)	0.30	0.88	0.21
SVM	0.97	835	0.63	(0.55, 0.70)	0.32	0.85	0.19
	With ROSE-sampling (Design 2)						
Method	AUC	Training Time (sec.)	AUC	95% CI for AUC	Sensitivity	Specificity	PPV
LR	0.74	3	0.63	(0.55, 0.70)	0.38	0.72	0.12
RF	0.74	33	0.67	(0.51, 0.76)	0.09	0.97	0.26
GBM	0.74	48	0.70	(0.61, 0.75)	0.09	0.98	0.45
XGBoost	0.76	2,340	0.74	(0.64, 0.78)	0.20	0.98	0.43
SVM	0.80	1343	0.68	(0.53, 0.76)	0.38	0.88	0.28
	With feature selection and ROSE-sampling (Design 3)						
Method	AUC	Training Time (sec.)	AUC	95% CI for AUC	Sensitivity	Specificity	PPV
LR	0.70	3	0.64	(0.56, 0.72)	0.53	0.69	0.15
RF	0.70	12	0.65	(0.56, 0.70)	0.30	0.89	0.24
GBM	0.69	30	0.66	(0.58, 0.74)	0.17	0.95	0.26
XGBoost	0.70	2,130	0.65	(0.56, 0.73)	0.17	0.95	0.27
SVM	0.71	811	0.65	(0.56, 0.70)	0.45	0.78	0.15

Table V. Confusion metrices for machine learning models

LR		RF		GBM		XGBoost		SVM	
No feature selection and sampling (Design 1)									
TP	FN	TP	FN	TP	FN	TP	FN	TP	FN
17	36	4	37	17	57	16	37	16	37
FP	TN	FP	TN	FP	TN	FP	TN	FP	TN
74	509	30	565	35	527	62	521	79	504
With ROSE-sampling (Design 2)									
TP	FN	TP	FN	TP	FN	TP	FN	TP	FN
24	40	4	39	5	50	10	40	23	37
FP	TN	FP	TN	FP	TN	FP	TN	FP	TN
160	412	11	582	6	575	13	573	62	514
With feature selection and ROSE-sampling (Design 3)									
TP	FN	TP	FN	TP	FN	TP	FN	TP	FN
30	27	18	42	8	40	8	40	28	39
FP	TN	FP	TN	FP	TN	FP	TN	FP	TN
172	407	58	518	23	565	22	566	133	436

Note: TP: True Positive, FP: False Positive, FN: False Negative, TN: True Negative.

Table VI. Distribution of NIHSS score before and after imputation.

**Distribution of NIHSS before Imputation**							
Train set				Test set			
0 to 4	5 to 11	12 to 23	Above 24	0 to 4	5 to 11	12 to 23	Above 24
281 (57%)	131 (27%)	61 (12%)	19 (4%)	80 (54.5%)	36 (24%)	24 (16%)	7 (5.5%)
**Distribution of NIHSS after Imputation**							
Train set				Test set			
0 to 4	5 to 11	12 to 23	Above 24	0 to 4	5 to 11	12 to 23	Above 24
1266 (50%)	733 (29%)	364 (14%)	185 (7%)	272 (43%)	201 (32%)	111 (17%)	52 (8%)

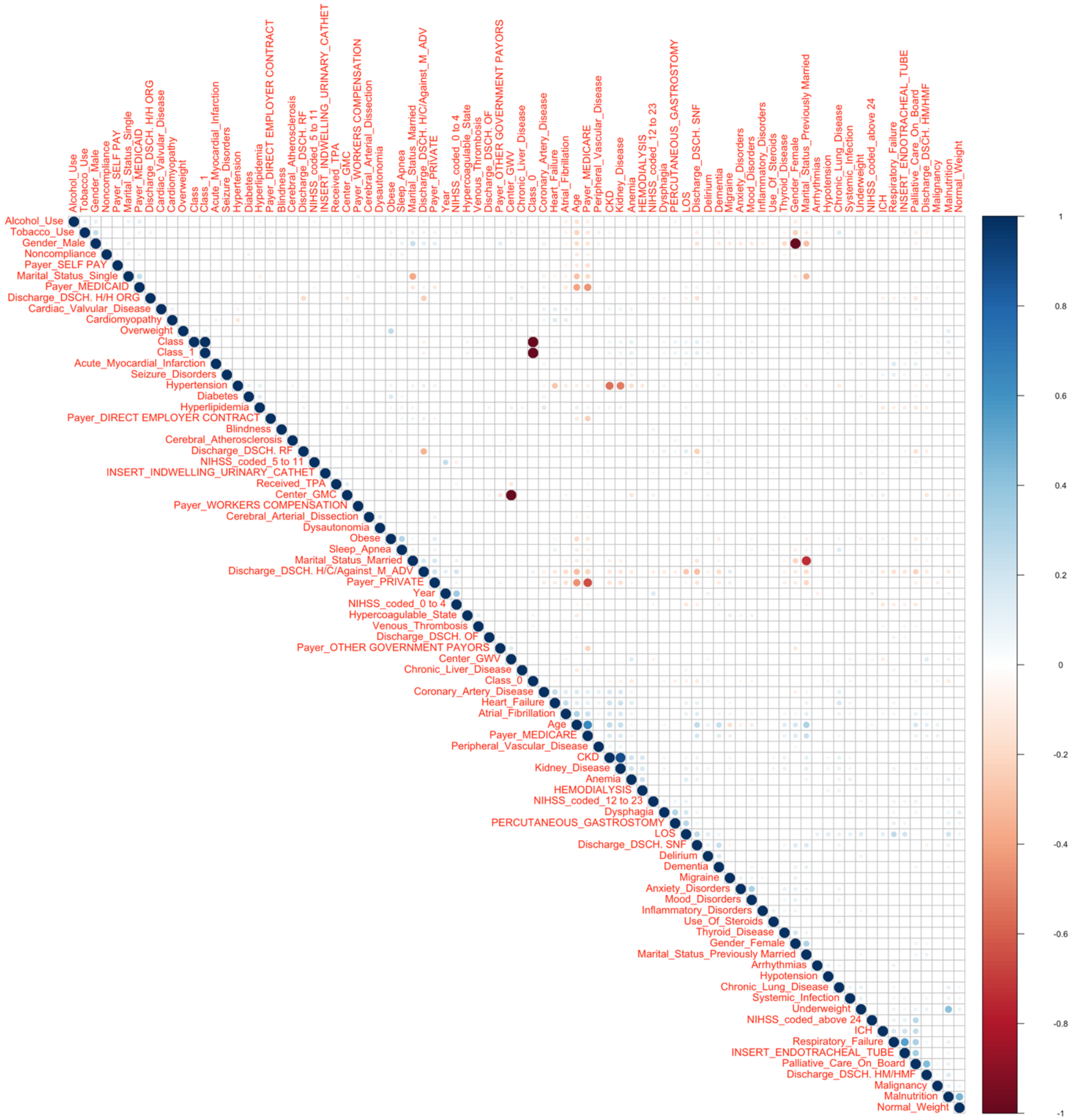

Figure I. Correlation matrix between all the variables.

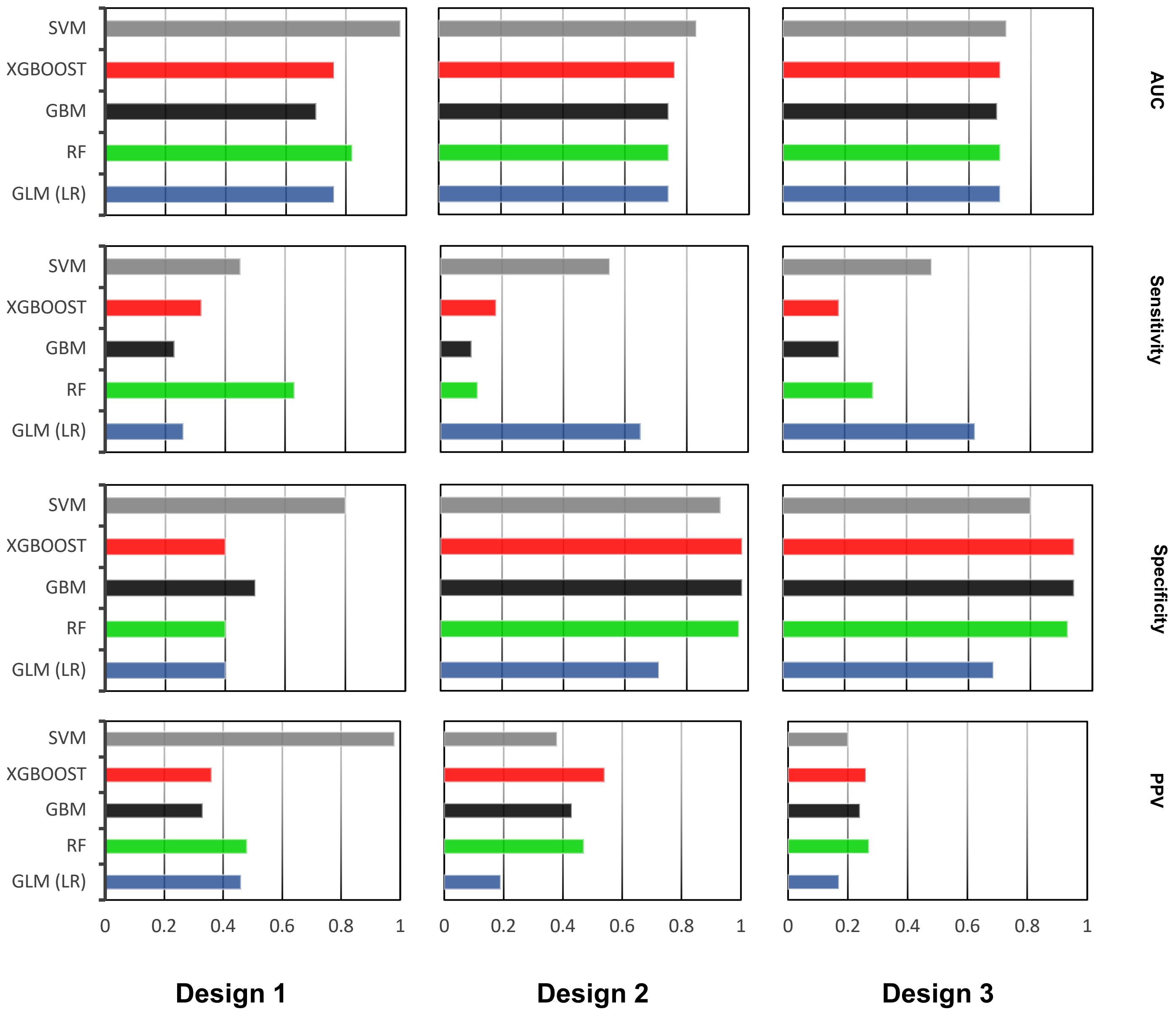

Figure II. Performance metrics of machine learning models for train sets.

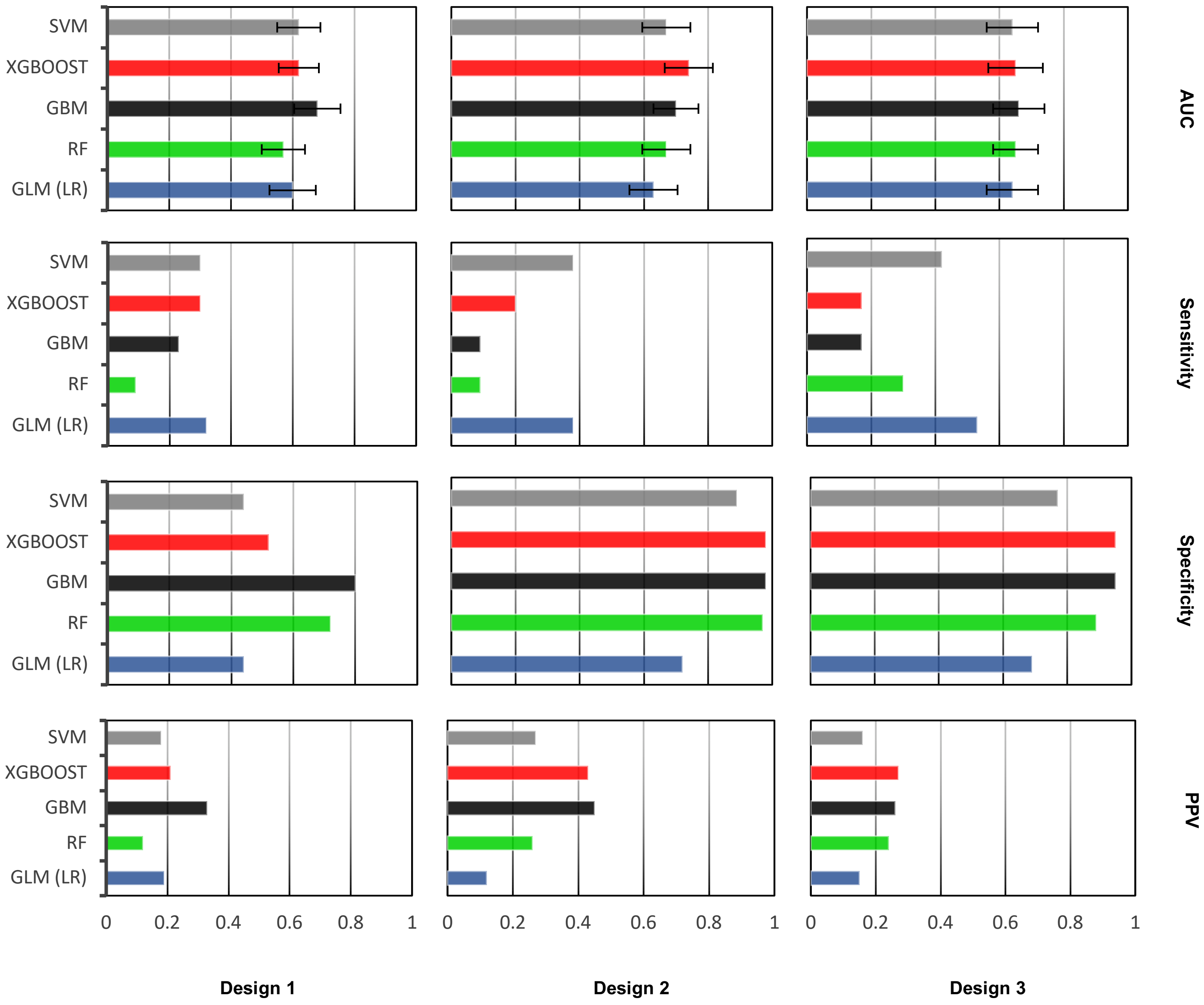

Figure III. Performance metrics of machine learning models for test sets (For AUC we provided 95% CI).

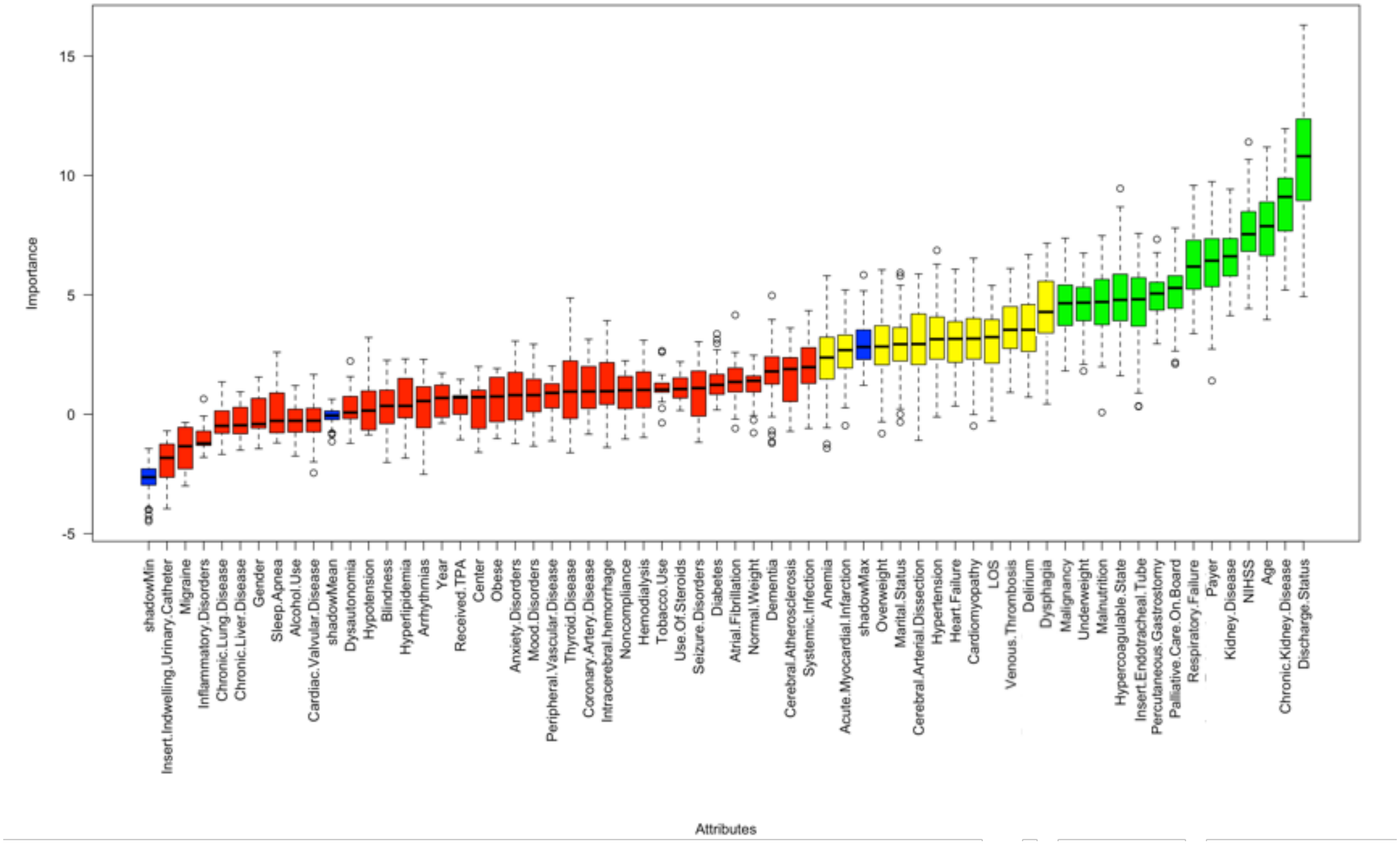

Figure IV. Selected features using Boruta Package.

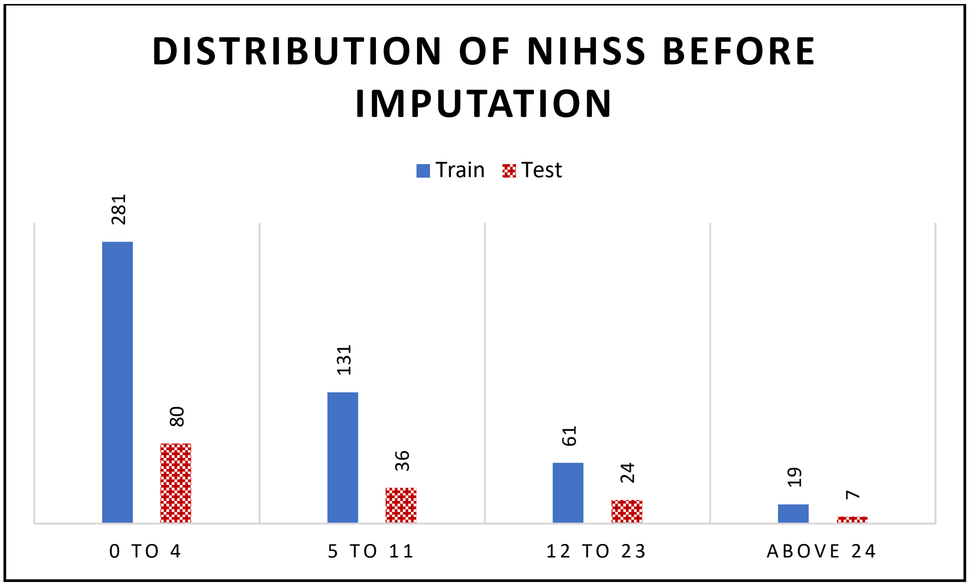

Figure V. Distribution of NIHSS before Imputation.

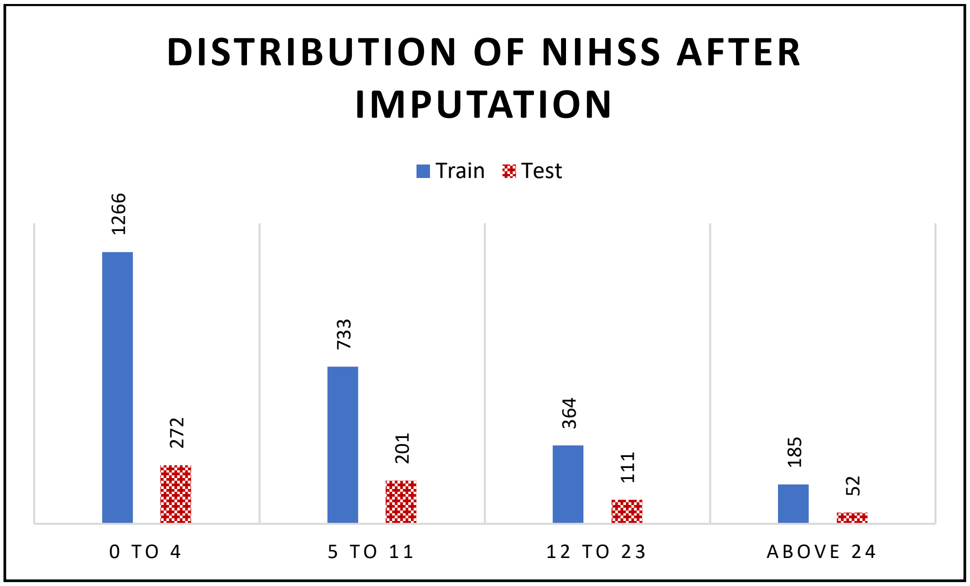

Figure VI. Distribution of NIHSS after Imputation.
